# Neuroendocrine neoplasms as a lynch syndrome manifestation: a case report and comprehensive literature review

**DOI:** 10.3389/fendo.2025.1587889

**Published:** 2025-06-17

**Authors:** Maria Paula Bernal Zárate, Daniel Felipe Mendivelso-Gonzalez, William Camilo Torres, Angelica Maria González Clavijo, Diego Felipe Ballen, Rafael Parra Medina, Julián C. Riaño-Moreno

**Affiliations:** ^1^ Department of Genetics, Fundación Universitaria de Ciencias de la Salud, Bogotá, Colombia; ^2^ Department of Pathology and Molecular Oncology, Instituto Nacional de Cancerología, Bogotá, D.C., Colombia; ^3^ Department of Endocrinology, Instituto Nacional de Cancerología, Bogotá, D.C., Colombia; ^4^ Faculty of Medicine, Universidad Cooperativa de Colombia, Villavicencio, Colombia; ^5^ Department of Clinical Oncology, Instituto Nacional de Cancerología, Bogotá, D.C., Colombia

**Keywords:** lynch syndrome, neuroendocrine neoplasms (NENs), mismatch repair-deficient (dMMR), neuroendocrine tumor (NET), hereditary cancer

## Abstract

Lynch syndrome (LS) is an autosomal dominant inherited disorder caused by pathogenic variants in DNA mismatch repair (MMR) genes, most commonly *MLH1* and *MSH2*. LS significantly increases the risk of various cancers, including colorectal, endometrial, gastric, and ovarian malignancies. Neuroendocrine neoplasms (NENs) are rare tumors that arise from neuroendocrine cells, predominantly in the gastrointestinal tract, and are frequently associated with hereditary cancer syndromes such as multiple endocrine neoplasia types 1 and 2. While a definitive association between LS and NENs has not been established, isolated cases have been reported. We present the case of a 63-year-old woman with a history of colorectal cancer and a confirmed LS diagnosis, identified through genetic testing that revealed a pathogenic *MLH1* variant. Years later, she developed a grade 2 non-functional neuroendocrine tumor (NET), likely of gastrointestinal origin. The patient underwent surgical resection, followed by treatment with somatostatin analogs. Due to this uncommon presentation, we conducted a literature review to explore the potential relationship between LS and NENs. Our analysis identified 13 additional cases of NENs in LS patients, encompassing NETs, neuroendocrine carcinomas (NECs), and mixed neuroendocrine non-neuroendocrine neoplasms (MiNENs). This growing body of evidence suggests that NENs may be part of the LS tumor spectrum. Further research is needed to elucidate the underlying mechanisms and determine whether LS predisposes individuals to NENs. Enhanced surveillance in LS patients could improve early detection of rare malignancies such as NENs, ultimately expanding our understanding of LS-associated cancer risks and guiding more effective clinical management.

## Introduction

Lynch syndrome (LS), also known as hereditary non-polyposis colorectal cancer (HNPCC), is an autosomal dominant disorder caused by pathogenic variants in DNA mismatch repair (MMR) genes, including *MLH1, MSH2, MSH6, PMS2*, and, occasionally, *EPCAM* (1). These genes play a critical role in maintaining genomic integrity by correcting DNA replication errors. *MLH1* and *MSH2* variants account for most LS cases, with *MLH1* pathogenic/likely pathogenic (P/LP) variants primarily associated with colorectal tumors, whereas MSH2 P/LP variants are linked to an increased risk of colorectal, endometrial, and ovarian cancers (2). LS is associated with a broad spectrum of malignancies, including colorectal (CRC), endometrial, gastric, ovarian, pancreatic, urothelial, biliary tract, and small intestinal cancers, as well as brain tumors (predominantly glioblastomas). Additionally, LS can manifest with sebaceous neoplasms (sebaceous adenomas, sebaceous carcinomas, and keratoacanthomas), particularly in Muir-Torre syndrome (3). Early identification of LS and genetic counseling are crucial for implementing targeted cancer prevention and surveillance strategies, improving patient prognosis and quality of life (4).

Neuroendocrine neoplasms (NENs) arise from neuroendocrine cells, which have both neural and endocrine properties, allowing them to respond to neuronal stimuli and secrete hormones or signaling molecules into the bloodstream. Due to the widespread distribution of neuroendocrine cells, NENs can develop in various organs, including the esophagus, stomach, pancreas, intestines, lungs, and endocrine glands (5). These tumors are characterized by the expression of neuroendocrine markers, such as synaptophysin and chromogranin A, and variable somatostatin receptor expression (6).

NENs are classified into three main categories: well-differentiated neuroendocrine tumors (NETs), poorly differentiated neuroendocrine carcinomas (NECs), and mixed neuroendocrine non-neuroendocrine neoplasms (MiNENs) (7). NETs are the most common and typically follow an indolent course, while NECs are highly aggressive. MiNENs combine a high-grade neuroendocrine component with a non-neuroendocrine malignancy, often exhibiting a poor prognosis. Most NETs (55%) originate in the gastrointestinal tract, with the small intestine being the most frequent site (45%), followed by the rectum (20%), appendix (16%), colon (11%), and stomach (7%) (8).

Approximately 20% of neuroendocrine tumors (NETs) are associated with hereditary cancer syndromes. The most well-established of these include multiple endocrine neoplasia types 1 and 2 (MEN1 and MEN2), as well as neurofibromatosis type 1 (NF1), Carney complex, Von Hippel-Lindau disease, and tuberous sclerosis complex, all of which are linked to an increased risk of NET development (9).

To date, no definitive association has been established between NENs and LS. These conditions are considered distinct clinical entities, characterized by different genetic alterations and pathogenic mechanisms. However, an increasing number of case reports have described NENs in patients with LS, suggesting a possible yet unconfirmed relationship.

In this study, we present a case of a NET in a patient with LS and conducted a literature analysis to explore the potential association between NENs and Lynch syndrome. Our review identified 13 additional cases with a clinical, molecular, or combined diagnosis of LS, including patients with NETs, NECs, and MiNENs. This is the first study to systematically compile the existing evidence on this association, highlighting the scarce but emerging data suggesting that NENs may be part of the LS tumor spectrum. However, due to the limited nature of the available evidence, further research is necessary to establish a definitive correlation.

## Materials and methods

### Case presentation

**Figure 1:**
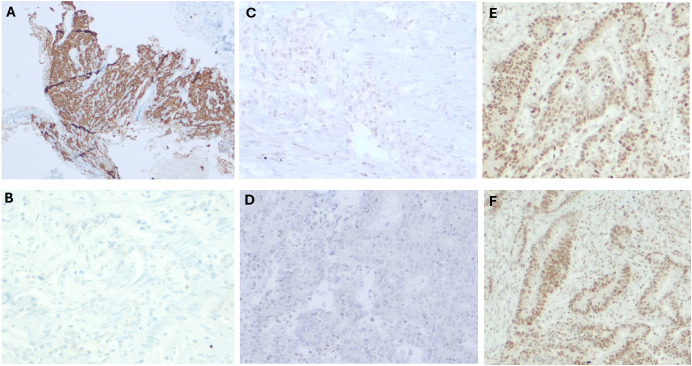
Immunohistochemical studies (40X) showing **(A)** Chromogranin IHC in the affected lymph node, showing a positive staining. **(B)** Chromogranin IHC from the rectosigmoid adenocarcinoma, showing a negative staining. MMR IHC from this tumor shows no expression of MLH1 **(C)** and PMS2 **(D)**, and an intact expression of MSH2 **(E)** and MSH6 **(F)**.

Our case describes a 50-year-old woman who presented to the emergency department in 2000 with hyporexia and postprandial liquid stools lasting three months, followed by an episode of acute abdominal pain requiring surgical exploration. Intraoperative findings revealed a colonic tumor mass, which was resected. She subsequently underwent eight cycles of adjuvant chemotherapy. Afterward, she was lost to follow-up until July 2015, when she was admitted to the Instituto Nacional de Cancerología in Bogotá, D.C. Colombia, at the age of 63 due to hematochezia. Colonoscopy identified a new lesion in the sigmoid colon, leading to a total colectomy with ileostomy and adjuvant chemotherapy. Pathological analysis confirmed a stage II rectosigmoid adenocarcinoma. Immunohistochemistry (IHC) demonstrated loss of MLH1 and PMS2 expression, with negative chromogranin staining (**Figure 1**). Molecular analysis of the rectosigmoid tumor by RT-PCR did not detect pathogenic variants in *BRAF, KRAS*, or *NRAS*.

**Figure 2:**
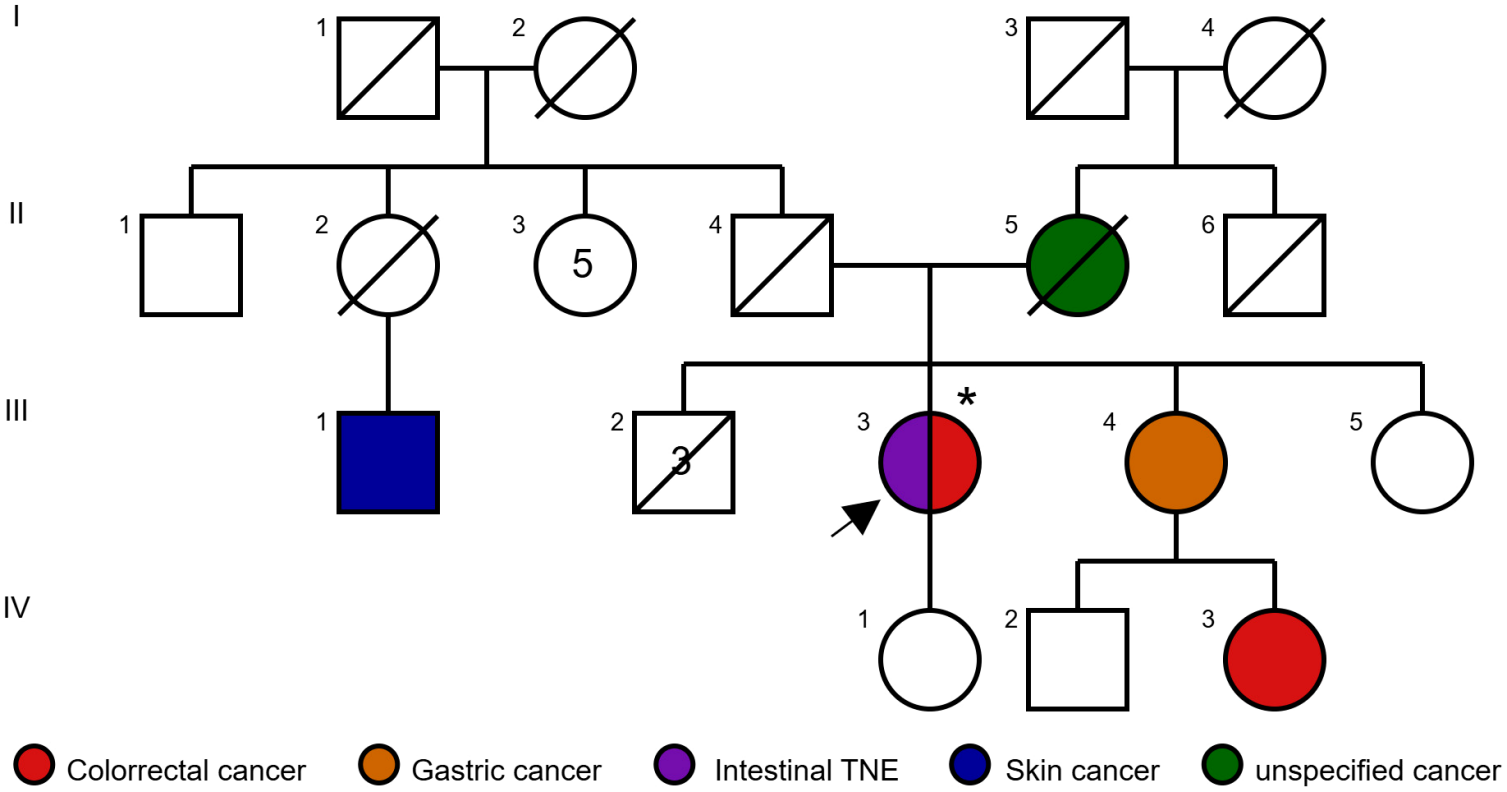
Pedigree of a family with Lynch syndrome, showing the proband with metachronous tumors (intestinal NET and colorectal cancer). *Documented carrier of pathogenic variant *MLH1*:c.1852_1854del; p.Lys618del.

Family history (**Figure 2**) revealed multiple first- and second-degree relatives with oncological conditions, including her mother (II-5), diagnosed with an unspecified malignancy at age 66; her sister (III-4), diagnosed with gastric cancer at age 63; and her niece (IV-3), diagnosed with CRC at age 38. Given her personal history of CRC and a family history of gastric and colorectal cancer, LS was suspected, and the patient was referred to the Medical Genetics Department at our institution for further evaluation.

Our patient met two out of five Bethesda criteria (1): CRC with high microsatellite instability (MSI-H) histology diagnosed before the age of 60 and (2) CRC or LS-associated tumors diagnosed at any age in at least two first- or second-degree relatives. She also met four out of five Amsterdam II criteria and fulfilled the National Comprehensive Cancer Network (NCCN) criteria for LS evaluation, which include clinical presentation in at least two generations, diagnosis of CRC or associated cancers before age 50, exclusion of familial adenomatous polyposis, and tumor verification through histopathology tests. Consequently, germline testing was performed using a Next-Generation Sequencing (NGS) multi-gene panel with the Illumina kit and the Canadian Consortia Inherited Cancer custom panel (Illumina, San Diego, CA), covering 105 genes associated with hereditary cancer syndromes, including mismatch repair (MMR) genes. Bioinformatic analysis was conducted using SOPHiA DDM^®^ software with the GRCh37/hg19 human genome reference. A pathogenic heterozygous MLH1 (NM_000249.4) variant (c.1852_1854del; p.Lys618del) was identified, with a variant allele fraction of 44.21% and an allelic depth of 252X, confirming the diagnosis of LS.

**Figure 3:**
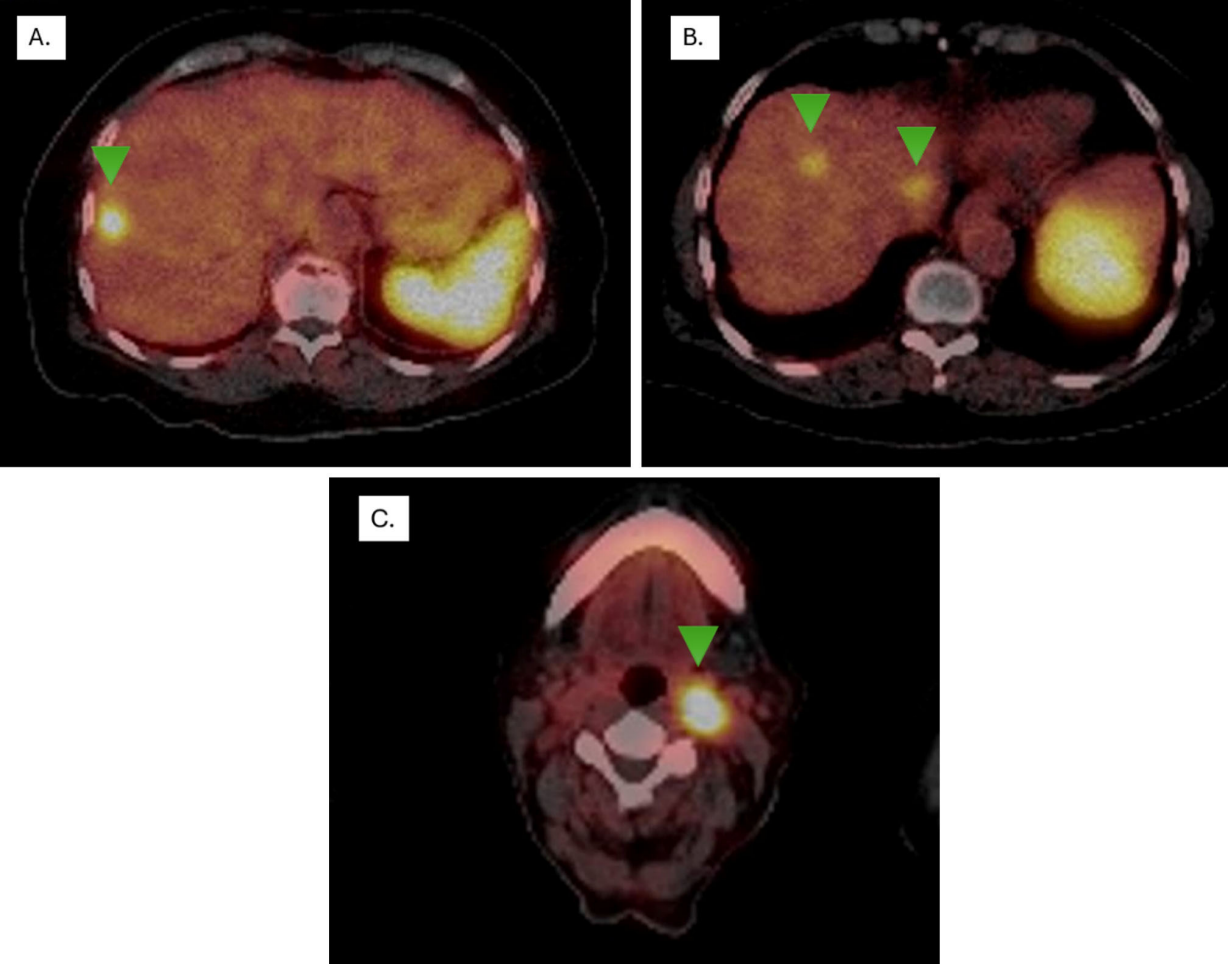
Ga-68 DOTA-péptido PETSCAN. Lesions with overexpression of somatostatin receptors (Green arrows) **(A)** segment VIII with a higher uptake, showing an SUVmax of 11.3 (previous study SUVmax 8.4), classified as Krenning 4, **(B)** segments VIII and I show SUVmax levels of 5.5 (previous study SUVmax 3.6) and 6.3 (previous study SUVmax 4.3), respectively, both classified as Krenning 3. **(C)** Lymph node in left station IIa, SUVmax of 40 (Krenning 4).

In December 2016, as part of LS and CRC follow-up, an abdominal MRI revealed three hypervascular focal lesions with dominant arterial phase enhancement, located in segment III (21 mm), segment VII (15 mm), and segment V (8 mm). These lesions were initially suspected to be hepatic metastases from the rectosigmoid adenocarcinoma (**Figure 3**). The patient underwent a hepatic metastasectomy, and histopathological analysis, along with IHC, confirmed metastatic involvement from a newly identified grade 2 non-functional NET, likely of intestinal origin. IHC showed positive staining for CDX2, synaptophysin, and chromogranin A, while CK7, CK20, and TTF1 were negative. The Ki-67 index was >4%, supporting the diagnosis.

Given this finding, a PET-CT scan using 68Ga-labeled somatostatin analogues (SSAs) was performed, revealing high somatostatin receptor expression in liver lesions and a left cervical lymph node (**Figure 1**). A biopsy of the lymph node confirmed NET infiltration, with IHC showing positive staining for chromogranin and synaptophysin (**Figure 3**). Based on the metastasectomy findings and PET-CT results, which guided a tru-cut biopsy at the cervical level and confirmed a grade II NET, first-line treatment with somatostatin analogues was initiated. Unfortunately, MMR status or MSI testing in the NET could not be performed due to insufficient material in the tissue block.

The patient remains under follow-up at our institution, with stable NET findings. However, in 2023, imaging revealed a hypervascular nodule adjacent to the resection margin of the previous hepatic metastasectomy, suggesting possible tumor recurrence. Given the clinical and imaging findings, a multidisciplinary medical board determined that the tumor had not progressed, and no additional invasive biopsies were indicated. The patient continues treatment with subcutaneous lanreotide 120 mg every 28 days. Aside from CRC, the patient has not developed any other LS-related tumors. Following the LS diagnosis, she underwent a risk-reducing hysterectomy with bilateral salpingo-oophorectomy in late 2023. Segregation studies of the *MLH1* germline variant could not be performed, as some family members were unreachable, while others declined testing.

### Literature analysis

A literature search was performed in MEDLINE, EMBASE, and Web of Science. The search included articles published between January 2000 to December 2024, using key terms including “Lynch Syndrome” OR “hereditary nonpolyposis colorectal cancer (HNPCC)” AND “neuroendocrine neoplasm” OR “NEN” OR Neuroendocrine Tumor” OR “NET.” Additionally, we included searches for “MMR deficiency” OR “MSI” AND “Neuroendocrine neoplasm” OR “NEN” OR Neuroendocrine Tumor” OR “NET.”

**Figure 4:**
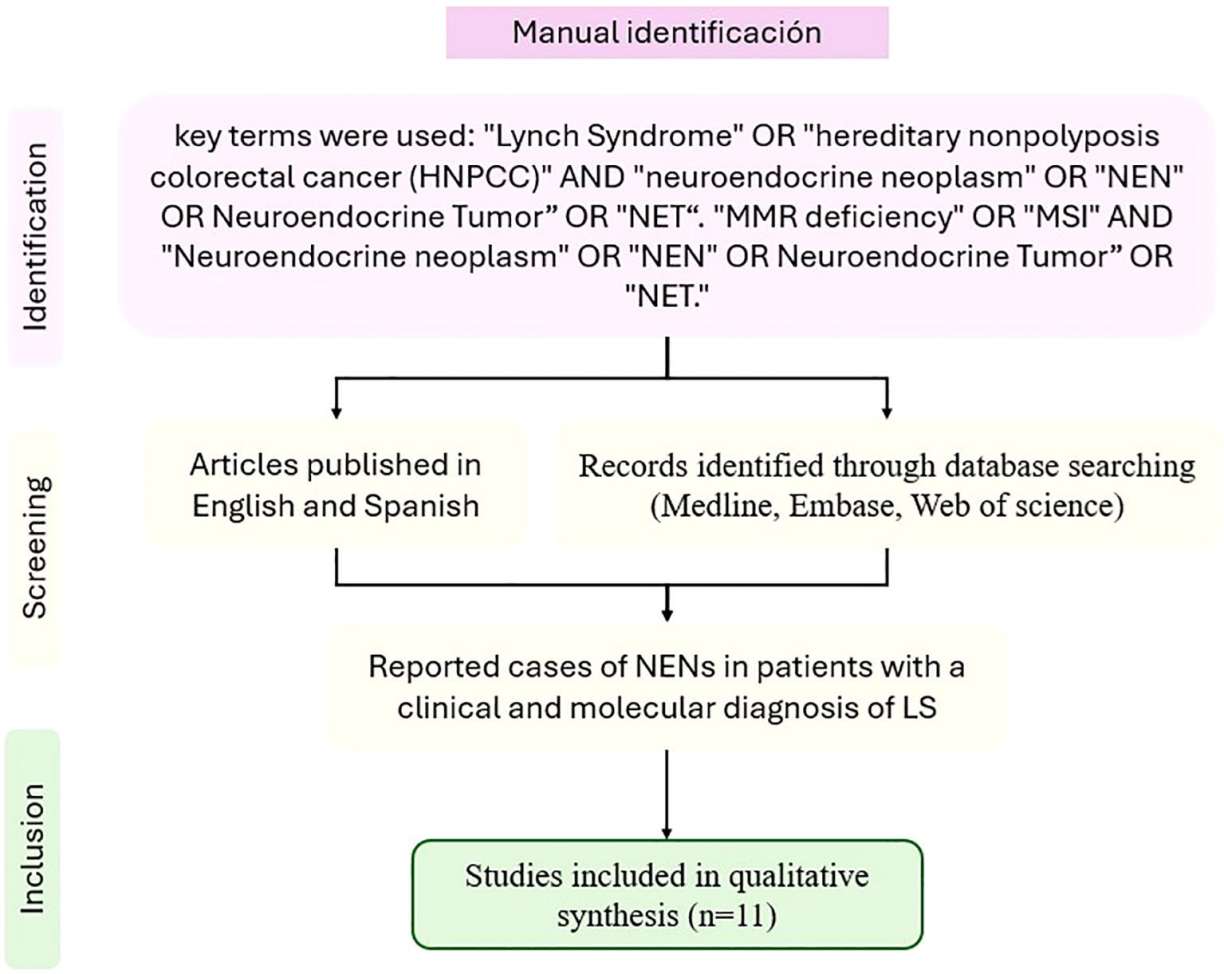
PRISMA flowchart for the manual literature review.

We included articles reporting cases of NENs in patients with a clinical and molecular diagnosis of LS. Articles published in English and Spanish were considered. Only duplicate articles across databases were excluded; no exclusions were made based on article type. Ultimately, 11 articles were included for full-text analysis (**Figure 4**). Data extraction was conducted by MPBZ and DFMG, while AMGC and JCRM resolved any conflicts regarding case inclusion or data extraction.

## Results and discussion

LS confers an elevated risk for a spectrum of cancers (10), a spectrum that has broadened with enhanced disease surveillance (11). Prospective long-term follow-up studies have identified additional cancer types, including gastric, urinary tract, pancreaticobiliary, small intestinal, breast, and brain cancer. However, robust evidence supporting the efficacy of systematic screening for these less common LS-associated malignancies remains limited, with specialized screening primarily guided by strong familial aggregation of specific cancers (12). While NENs are not traditionally considered within the LS-associated cancer spectrum (3), we present a case of LS manifesting with metachronous rectosigmoid adenocarcinoma and a gastrointestinal NEN. This unusual presentation prompted a comprehensive literature review and analysis to further explore the potential biological and clinical relationship between LS and NENs.

Our literature review identified ten case reports and one case series, documenting thirteen unrelated cases (**Table 1**) describing the association between LS and NENs (13–17, 19–23) (summarized in **Table 2**). This cohort exhibited an equal sex distribution (50% female, 50% male). The mean age at first tumor presentation was 46.4 years, while the mean age at NEN diagnosis was 56.4 years. Of the fourteen individuals (including our case), seven received an LS diagnosis before the age of 50; however, only four developed a NEN at a similarly young age.

**Table 1:** Description of case reports on patients with neuroendocrine neoplasms associated with Lynch syndrome.

Study Reference, Country report	Total Reported Cases	NEN type	Patient Gender	Criteria: Amsterdam II; Bethesda	Age at Diagnosis (yrs)	Tumor Type(s)	MMR Status by IHC				Additional Histopathological Findings	MSI Status	Germline Testing Method	Germline Variant Gene/cDNA/Protein†
							MLH1	MSH2	MSH6	PMS2				
Our case, Colombia	1	NET	F	4/5; 2/5	57	Sigmoid colon adenocarcinoma	(-)	(+)	(+)	(-)	NR	NR	NGS	*MLH1*:c.1852_1854del; p.Lys618del
					58	NET in liver and cervical lymph node metastases, possible primary small intestine	NR	NR	NR	NR	CDX2 (+), synaptophysin (+), chromogranin A (+), CK7 (-), CK20 (-), TTF1 (-)			
Wagner et al., 2010, USA(13)	1	MiNEN	M	5/5; 5/5	41	Moderately differentiated adenocarcinoma of the colon	NR	NR	NR	NR	NR	NR	NR	*MSH2* not informed
					65	High-grade urothelial carcinoma of the left renal pelvis/kidney	NR	NR	NR	NR	NR			
					66	Carcinoma *in situ* of the bladder	NR	NR	NR	NR	NR			
					73	Poorly differentiated adenocarcinoma of the small bowel	NR	NR	NR	NR	NR			
					77	Moderately differentiated adenocarcinoma of the colon	NR	NR	NR	NR	NR			
					78	High-grade urothelial carcinoma of the right ureter	NR	(-)*	NR	NR	NR			
					78	High-grade prostatic intraepithelial neoplasia was identified with neuroendocrine (small cell) (HGPIN-NE)	NR	(-)*	NR	NR	Chromogranin (+)			
Sorscher et al., 2013, USA(14)	1	NET	F	NR; 1/5	52	Colon adenocarcinoma,					NR	NR	NR	*MLH1*:c.1853del; p.Lys618Argfs*19
					57	Gastric adenocarcinoma	(-)*	NR	NR	NR	NR			
					63	High-grade hepatic NET	(-)*	NR	NR	NR	Synaptophysin (+), pankeratin (+), CD56 (+), chromogranin (+)			
Karamurzin et al., 2012, USA (17)(15)	1	NET	F	5/5; 4/5	46	Endometrial carcinoma, Colonic villous adenoma with high-grade dysplasia	(+)	(-)	(-)	(+)	NR	NR	NR	NR
					48	Pancreatic NET	(+)	(-)	(-)	(+)	Synaptophysin (+), chromogranin (+), trypsin (-), chymotrypsin (-).			
Yousef et al., 2014, USA(16)	1	NEC	F	3/5; 3/5	34	Cervical small cell NEC	(-)*	NR	NR	NR	Synaptophysin (+), common leukocyte antigen (-), chromogranin (-)	NR	NR	NR
Kidambi et al., 2017, USA(17)	3	NEC	M	3/5; 2/5	62	Poorly differentiated large cell NEC of the colon	(+)	(+)	(-)	(+)	Cam5.2 (+), synaptophysin (+), chromogranin (+)	NR	NGS + CNVs	*MSH6*:c.1634_1637del; p.Lys545Argfs*25
		NEC	M	3/5; 2/5	47	Sigmoid adenocarcinoma	NR	NR	NR	NR	NR		NGS	*MLH1*:c.1456dup; p.Ser486Phefs*17
					60	Colonic NEC composite plus adenocarcinoma	(-)	(+)	(+)	(-)	Synaptophysin (+), chromogranin (+)			
		NET	M	3/5; 2/5	50	Cecal adenocarcinoma	NR	NR	NR	NR	NR		NR	*MLH1* (deletion of exons 3–6)
					50	Rectal adenocarcinoma	NR	NR	NR	NR	NR			
					57	Ascending colon adenocarcinoma	NR	NR	NR	NR	NR			
					57	Well-differentiated NET of the colon	(-)	(+)	(+)	(-)	Synaptophysin (+), chromogranin (+)			
Serracant et al., 2017, Spain(18)	1	NET	F	5/5; 5/5	45	Descending colorrectal carcinoma	NR	NR	NR	NR	NR	MSI-H	NR	*MLH1*:c.731G > A; p.Gly244Asp
					57	Ascending colorrectal carcinoma	NR	NR	NR	NR	NR			
					58	Endometrial adenocarcinoma	(-)	(+)	(+)	(-)	NR			
					63	Invasive right low-grade dulcal adenocarcinoma	NR	NR	NR	NR	NR			
					65	Low-grade duodenal adenocarcinoma	(-)	(+)	(+)	(-)	NR			
					65	Sebomatricoma					NR			
					65	Pancreatic head NET	(-)	(+)	(+)	(-)	NR			
Whitman et al., 2019, USA(19)	1	MiNEN	M	NR; 1/5	60	Colon adenocarcinoma with large cell neuroendocrine carcinoma component	(-)	(+)	(+)	(-)	Synaptophysin (+), chromogranin (+)	MSI-H	NR	*MLH1*:c1456dupT; p.Ser486Phefs*17
Dawley et al., 2021, USA(20)	1	MiNEN	M	NR; 2/5	NR	Unspecified colon cancer	NR	NR	NR	NR	NR	MSI-H	NGS	*MSH6*:c.3 491dupT; p.Cys1165Valfs*7
					NR	Melanoma	NR	NR	NR	NR	NR			
					80	Poorly differentiated gastric adenocarcinoma (30% large cell NET, 70% adenosquamous carcinoma)	(+)	(+)	(-)	NR	Adenocarcinoma: CK7(+), p40 (+); NET: Synaptophysin (+), CD56 (+), chromogranin A (+) MSH6 (+) PMS2 (+) MSH2 (+)			
Kobayashi et al., 2022, Japan(21)	1	NET	M	4/5; 3/5	29	Cecal not specified cancer, Adenocarcinoma of the rectum, ascending and sigmoid colon	(+)	(-)	(-)	(+)	NR	MSI-H	NR	NR
					44	NET of the descending colon	(+)	(-)	(-)	(+)	Chromogranin A (+), synaptophysin (+), CD56 (+), CK7 (-), CK20 (+)			
Brignola et al., 2024, Italy(22)	1	NET	F	3/5; 2/5	26	Poorly differentiated cecal adenocarcinoma	(-)	(+)	(+)	(-)	NR	NR	NGS	*MLH1*:c.(453 + 1_454-1)_(545 + 1_546-1)del; p.Glu153Phefs*8
					26	Well-differentiated NET of the appendix	(+)	(+)	(+)	(+)	NR			
Siu et al., 2023, USA(23)	1	MiNEN	F	4/5; 2/5	55	Poorly differentiated endometrial adenocarcinoma (80% large cell neuroendocrine tumor, 20% endometrioid adenocarcinoma)	NR	NR	(+)	(-)	Estrogen receptor (moderate +), progesterone receptor (moderate +), CD56 (focal +), vimentin (focal +), p53 (+, wild type), ki67 (+, 90%), MSH6 (+), PMS2 (-), p63 (-), p16 (-), chromogranin (-), synaptophysin (-)	NR	NR	NR

* No IHQ specified, just the loss of function of one protein, † HGVS (Human Genome Variation Society) nomenclature; M, Male; F, Female; CDX2, Caudal Type Homeobox 2; CK, Cytokeratin; CNVs, Copy Number Variants; HGPIN-NE, High-Grade Prostatic Intraepithelial Neoplasia with Neuroendocrine Features; IHC, Immunohistochemistry; MiNEN, Mixed neuroendocrine non-neuroendocrine neoplasms; MMR, Mismatch Repair; MSI, Microsatellite Instability; NGS, Next-Generation Sequencing; NEC, Neuroendocrine Carcinoma; NEN, Neuroendocrine Neoplasm; NET, Neuroendocrine Tumor; NR, Not Reported; PCR, Polymerase Chain Reaction; TTF1, Thyroid Transcription Factor 1; USA, United States of America.

**Table 2:** Summary of reported cases of neuroendocrine neoplasms in Lynch syndrome patients.

Category	Total	NEN Type		
		NEC	MiNEN	NET
Total Cases (n)	14	3	4	7
Men (M)	7	2	3	2
Female(F)	7	1	1	5
Age of initial cancer diagnosis (yrs)				
Mean and range (min-max)	46,4 (26-60)	47,6 (34-62)	52 (41-60)	43,5 (26-57)
Diagnostic criteria met (n)				
Amsterdam criteria met (min-max)	3-5	3	4-5	3-5
Bethesda criteria met (min-max)	1-5	2-3	1-5	1-5
NEN location (n)				
Colonic	6	2	1	3
Appendix	1	0	0	1
Prostate	1	0	1	0
Hepatic	1	0	0	1
Pancreatic	2	0	0	2
Gastric	1	0	1	0
Cervix	1	1	0	0
Endometrial	1	0	1	0
NEN MMR by IHC (n)				
MLH1	6	2	1	3
MLH2	3	0	1	2
MSH6	4	1	1	2
PMS2	5	1	2	2
MSI Status (n)				
MSI-H	4	0	2	2
Germline Testing identified genes (n)				
*MLH1*	7	1	1	5
*MSH2*	1	0	1	0
*MSH6*	2	1	1	0

All reported cases fulfilled at least one Bethesda criterion, although Amsterdam II criteria could not be fully assessed in two cases due to insufficient clinical information. Only three cases met all Amsterdam II criteria, while two cases satisfied all Bethesda criteria. Consequently, all cases identified can be considered LS-related based on established clinical guidelines. Notably, in three out of fourteen cases (21.4%), the NEN was the initial malignancy detected, preceding the diagnosis of LS.

The majority of reported NENs (11/14) originated within the gastrointestinal tract, with the colon being the most frequent primary site (6/11), followed by the pancreas (2/11), liver (1/11), stomach (1/11), and appendix (1/11). In our case, the NEN likely arose from an intestinal primary site. Other less common primary sites included the prostate and cervix, with presentations occurring either synchronously or metachronously with other LS-related tumors.

IHC analysis results for all four MMR proteins were available in only ten of the fourteen cases. Loss of MLH1 and PMS2 expression was the most frequent alteration (6/10 cases), followed by loss of MSH2 and MSH6 expression (2/10 cases). One case exhibited isolated MSH6 deficiency (a MiNEN), confirmed by germline molecular analysis, while another showed PMS2 loss (a MiNEN) without confirmatory somatic or germline studies. In the remaining cases, a single affected protein was reported without specifying the others, including MLH1 (2 cases) and MSH6 (1 case). Microsatellite instability (MSI) status was assessed in only four out of fourteen cases (two NETs and two MiNENs), all of which demonstrated high-frequency MSI (MSI-H).

The inherent heterogeneity of NENs complicates the establishment of direct and predictive correlations between MMR/MSI status, the specific molecular profile, and the NEN type. In **Table 1** display seven cases exhibit an IHC dMMR pattern consistent with the molecular profile identified through germline genetic testing: (i) a high-grade hepatic NET with dMMR (MLH1) and a pathogenic germline variant in the *MLH1* gene, (ii) a poorly differentiated colonic NEC with dMMR (MSH6) and a pathogenic germline variant in the *MSH6* gene, (iii) a colonic NEC with dMMR (MLH1/PMS2) and a pathogenic germline variant in the *MLH1* gene, (iv) a well-differentiated colonic NET with dMMR (MLH1/PMS2) and a pathogenic germline variant in the *MLH1* gene, (v) a pancreatic NET with dMMR (MLH1/PMS2) and a pathogenic germline variant in the *MLH1* gene, (vi) a colonic NEC with dMMR (MLH1/PMS2) and a pathogenic germline variant in the *MLH1* gene, and (vii) a gastric NEN with dMMR (MSH6) and a pathogenic germline variant in the *MSH6* gene. The observed correspondence between the IHC dMMR pattern and the germline molecular profile in these seven cases suggests the hypothesis that a portion of the pathogenic mechanisms underlying the development of a subgroup of NENs might be intrinsically linked to the pathophysiology of LS.

Regarding histology, seven out of fourteen cases were NETs, three were NECs, and four were classified as MiNENs, which included: high-grade prostatic intraepithelial neoplasia with neuroendocrine features (HGPIN-NE), poorly differentiated endometrial adenocarcinoma (80% large cell NET, 20% endometrioid adenocarcinoma), poorly differentiated gastric adenocarcinoma (30% large cell NET, 70% adenosquamous carcinoma), and colon adenocarcinoma with a large cell neuroendocrine carcinoma component. Notably, all NECs and MiNENs exhibited dMMR.

Several studies on non-LS-related NENs have indicated that dMMR/MSI-H is relatively common in colorectal NECs. MSI has been reported in three out of ten gastrointestinal NECs, with a prevalence ranging from 12.4% (24) to 30% (25). The most frequent finding is the loss of MLH1 and PMS2 expression, whereas dMMR/MSI-H remains highly uncommon in NENs originating from other anatomical sites. Interestingly, dMMR/MSI-H NECs exhibit distinct clinical behavior; none of the reported cases presented with distant metastases at diagnosis, and all were classified as stage II or III based on nodal involvement. The pathogenesis, clinicopathologic features, and molecular profiles of MSI-H NECs closely resemble those described for sporadic MSI-H adenocarcinomas of the stomach and colorectum.

Similar to MSI-H NECs, MSI-H colonic NETs exhibit pathogenetic and molecular characteristics comparable to MSI-H colonic adenocarcinomas (26). This similarity suggests that MSI-H colonic NETs may also benefit from early initiation of immune checkpoint inhibitor therapy. Although MMR status and MSI assessment could not be performed in our patient due to insufficient tissue material, the clinical presentation strongly suggests an MSI-H colonic NET phenotype, mimicking the behavior of MSI-H adenocarcinomas. Recognizing NENs as potential LS-related tumors could support the inclusion of MMR/MSI testing in clinical guidelines, facilitating early identification and tailored treatment approaches for LS patients. This would open new therapeutic avenues, with immunotherapy emerging as a promising strategy in this context.

Colorectal NECs and MiNENs are generally associated with poor prognosis (27). However, MSI or dMMR status has been linked to improved outcomes, likely because these tumors exhibit molecular and clinical behavior like MSI-positive adenocarcinomas (27). Due to this, some authors recommend routine IHC assessment of MMR proteins or MSI analysis in early-stage colorectal NECs and MiNENs to identify a subset of dMMR tumors with a significantly more favorable prognosis (28). Such testing should be conducted at the initial diagnosis to avoid delays in treatment decisions upon disease progression.

Given that colorectal NECs have a response rate of less than 50% to platinum-based chemotherapy and an overall survival of less than nine months, MSI testing at diagnosis could enable early initiation of immune checkpoint inhibitor therapy, mirroring treatment approaches used for MSI-H adenocarcinomas, which have shown clinical benefits in this setting (29).

In contrast, MSI is highly infrequent in non-LS-related NETs. However, studies analyzing larger tumor cohorts have reported MSI in 25.8% of colonic NETs and 13.3% of pancreatic NETs. It is important to note that these studies did not distinguish between patients with or without LS based on clinical or molecular criteria. In our review, dMMR was identified in six out of seven NETs (85.7%), with only one case being MMR-proficient (1/7), suggesting that LS could be associated with a distinct NET phenotype (18, 30).

Although our case and the other thirteen cases included in this analysis met the clinical criteria for LS, germline testing for MMR genes was reported in only ten out of fourteen cases. Specifically, five out of seven NETs (71.4%), three out of four MiNENs (75%), and two out of three NECs (66.7%) had available germline testing data. The most frequently affected gene was *MLH1*, identified in seven out of ten cases. These *MLH1* variants included four deletions (three frameshift and one in-frame, the latter predicted to be pathogenic), two frameshift duplications, and one pathogenic missense variant. Additionally, two cases involved *MSH6* frameshift variants (one duplication and one deletion), while one case reported a pathogenic variant in *MSH2*, though no further details were provided in the original report (**Table 2**). Notably, MiNEN and NEC cases exhibited heterogeneous germline results, with P/LP variants detected in *MLH1, MSH2*, and *MSH6* (**Table 2**). In contrast, *MLH1* variants were found in all tested NET cases, suggesting a potential genetic distinction between NETs and other NEN subtypes in LS.

Although the association between LS and NENs is uncommon, several mechanisms have been proposed to explain their potential relationship. First, P/LP variants in MMR genes, which define LS, lead to MSI. MSI, a hallmark of many LS-associated cancers, results in the accumulation of mutations throughout the genome, potentially increasing the risk of various malignancies, including rare tumors such as gastrointestinal NENs (31). Second, epigenetic alterations, such as promoter hypermethylation of tumor suppressor genes, are well-documented in LS-associated cancers. Similar epigenetic mechanisms may contribute to NEN development (32, 33). Lastly, individuals with LS may have an increased susceptibility to environmental carcinogens due to their deficient DNA repair system, potentially leading to the development of malignancies outside the classical LS spectrum, including NENs, particularly NETs (25, 34).

This evidence presents plausible mechanisms linking LS and NEN development, drawing upon established LS pathophysiology. The proposed mechanisms MSI-driven mutagenesis, epigenetic dysregulation, and increased susceptibility to environmental carcinogens, offer a biologically plausible rationale for this rare association. In our case, the presence of a P/LP variant in an MMR gene, confirming the LS diagnosis, directly supports MSI-driven mutagenesis. Determining the MSI status of both the adenocarcinoma and the NET in our case would further strengthen this link. While not investigated here, epigenetic alterations and environmental exposures remain potential contributing factors in LS-associated NENs. The metachronous presentation of two distinct tumor types within the LS spectrum, including a rare NEN, underscores the complex and potentially expanded tumor predisposition in individuals with germline MMR defects, highlighting the importance of comprehensive molecular characterization and long-term surveillance in LS patients, even for seemingly unrelated malignancies.

## Conclusions

In the present study, we describe an unusual case of LS manifesting with metachronous tumors, specifically a rectosigmoid adenocarcinoma and a non-functional NET likely of gastrointestinal origin, which we compare with a compilation of reported cases of LS patients with NENs. To our knowledge, this represents the most extensive compilation of cases to date exploring the potential relationship between LS and NENs. Although the available data remain limited and primarily derived from isolated case reports, our clinical, pathological, and molecular analysis provides preliminary evidence supporting this possible association, albeit revealing significant heterogeneity in the presentation of NENs in patients with LS.

Our findings prompt us to suggest the incorporation of MMR/MSI testing in the analysis of patients with NENs, particularly in those with a family history of cancer suggestive of LS (meeting clinical criteria and/or with genetic confirmation). Furthermore, we propose considering the inclusion of clinical evaluation and, when appropriate, imaging studies directed at the detection of NENs in the surveillance protocol for patients with LS. Enhanced surveillance strategies for NENs, such as pNETs, may incorporate advanced imaging, including contrast-enhanced computed tomography (CECT) and apparent diffusion coefficient (ADC) with normalized values (35, 36).

The co-occurrence of NENs and LS, while rare, suggests a potential shared molecular pathway, at least in a subset of cases, influenced by dysfunction of the MMR system. The observed molecular similarities between dMMR/MSI-H gastrointestinal NENs and adenocarcinomas with the same profile, concerning prognosis and response to immunotherapy, warrants the prospective consideration of MMR/MSI status as a biomarker with prognostic, therapeutic, and predictive value in LS patients who develop NENs.

Despite the limited number of NEN cases documented in the literature within the context of LS, which hinders the identification of a definitive shared molecular pathway, this study constitutes a fundamental starting point for future investigations aimed at elucidating the nature of this interconnection and exploring potential causality. Large-scale prospective studies are necessary to investigate the prevalence of NENs in patients with LS and vice versa, as well as to characterize the specific molecular profile of these tumors and their potential response to targeted therapies, including immunotherapy.

## Data Availability

The datasets presented in this study can be found in online repositories. The names of the repository/repositories and accession number(s) can be found in the article/supplementary material.
